# Biocompatibility of nanosilver-coated orthodontic brackets: an in vivo study

**DOI:** 10.1186/s40510-016-0152-y

**Published:** 2016-12-05

**Authors:** Gamze Metin-Gürsoy, Lale Taner, Emre Barış

**Affiliations:** 1 Department of Orthodontics, Faculty of Dentistry, Gazi University, Bişkek cad. 1. Sok. No: 4 06510, Emek, Ankara, Turkey; 2 Department of Oral Pathology, Faculty of Dentistry, Gazi University, Bişkek cad. 1. Sok. No: 4 06510, Emek, Ankara, Turkey

**Keywords:** Nanosilver, Biocompatibility, Bracket, Biomaterial science, In vivo

## Abstract

**Background:**

Nanosilver particles of which antibacterial and antifungal properties have been shown in various in vitro and in vivo studies are used in many medical and dental fields for the prevention of infection. In this study, it is intended to evaluate the biocompatibility of nanosilver-coated brackets.

**Methods:**

Nanosilver coating process was applied to the standard orthodontic brackets by a physical vapor deposition system. Brackets were coated with nanosilver particles of 1 μ thickness. A total of 12 Wistar Albino rats were included in the study (six) and control (six) groups. For the study and control groups, four nanosilver-coated and four standard brackets were aseptically implanted subcutaneously in the dorsal region of each rat. The brackets were removed with the surrounding tissues on days 7, 14, 30, and 60. The specimens were evaluated for inflammatory response.

**Results:**

No significant difference was found in terms of tissue reaction between the study and control groups. On day 7, randomly distributed brown-black granules were seen in the granulation tissue adjacent to the bracket in the study group. These foreign particles continued along the bracket cavity in a few samples, but the inflammatory response was insignificant between the groups. Mast cell count was found to be significantly smaller only on day 7 in the study group than in the control group.

**Conclusions:**

Nanosilver-coated orthodontic brackets were found to be similar with the standard type concerning inflammation. Further researches are needed with regard to the assessment of the brown-black granules, especially on the deposition of the vessel walls.

## Background

Silver and silver compounds are known as strong antibacterial agents and have been used in various medical applications since ancient times. Marion Sims and Carl Siegmund Franz Crede used silver for the treatment of vesicovaginal fistulas by silver suture and prevented gonorrheal ophthalmia in newborn infants by silver nitrate solution, respectively [1]. After the discovery of antibiotics, the use of silver has lost its popularity. However, in the last few decades, researchers have started looking for new antibacterial agents due to multidrug-resistant bacteria, which is one of the biggest problems of antibiotic use. With recent advances in researches, nanotechnology has gained superior attention in antimicrobial properties of silver by using silver in the form of nanoparticles [2–4]. Nanoparticles are clusters of atoms in the size range of 1–100 nm and have outstanding chemical, optical, and mechanical features [5]. Currently, nanosilver particles have been applied to a wide range of health-care products, such as burn dressings, water purification systems, and dental and medical devices [6–15].

Irreversible, unhealthy and unaesthetic enamel demineralization (WSL) is the most common side effect of fixed orthodontic treatment. The nanoparticles show efficient antimicrobial properties due to their extremely large surface area, which provides better contact with microorganisms. Researchers reported that orthodontic brackets coated with nanoparticles or combining dental material with nanoparticles show antibacterial and anti-adhesive properties against normal oral pathogenic bacteria [15, 16].

The purpose of orthodontic treatment with fixed appliances is to improve function and esthetics. Although direct bonding, orthodontic attachments are excellent devices, their most common side effect is white spot lesion during treatment due to the failure of cleaning the teeth properly. Shortly after the start of the treatment, bacterial plaque comprising *Streptococcus mutans* may rapidly accumulate around the brackets and orthodontic bands [17–20].

Nanosilver particles of which antibacterial and antifungal properties have been shown in various in vitro and in vivo studies are used in many medical and dental fields in order to prevent infection [8, 13, 21–23]. Nanosilver biocompatibility is a controversial issue. There are many in vitro studies which show nanosilver particles to be toxic [24, 25] or nontoxic [26–28]. Cytotoxicity tests of medical materials prepared with the addition of nanosilver particles showed them as nontoxic [21–23, 29, 30]. Biocompatibility of a new material and medical devices must be analyzed before their use in humans. There are many test methods for the assessment of biocompatibility, and they can be divided into three categories: in vitro, animals, and usage test. Implantation tests involve animal tests and biocompatibility of new materials or medical device evaluation based on whether they contact with the bone or subcutaneous tissue.

The aim of the present study was to evaluate the biocompatibility of nanosilver-coated brackets, which can be used in the human teeth for the reduction of the areas of tooth decay and demineralization during orthodontic treatment with the advantages of the antibacterial properties of nanosilver, and thus to create a new type of bracket in the field of orthodontics.

## Methods

All experiments were approved by the Animal Experimentation Committee at Gazi University (research project number: G.Ü.ET-12.015) and conformed to the ARRIVE guidelines for animal research [31] and Rules for Animal Experiments of Gazi University. The sample size (*n* = 6) per group was determined depending on the decision of the ethics committee. All animal experiments were performed by one researcher (GMG) who had a certificate according to the guidelines for the proper conduct of animal experiments. The rats were kept under an artificial 12-h light/darkness cycle. Lights were turned on at 7 a.m. and off at 7 p.m. Room temperature varied between 22 and 24 °C, and appropriate room ventilation was maintained. Histological analyses were performed at Gazi University, Faculty of Dentistry, Department of Oral Pathology.

### Coating procedure

Mandibular premolar orthodontic brackets (Gemini Roth; 3M Unitek, Monrovia, CA, USA) were used in this study. All brackets were cleaned sonically with alcohol for 15 min. The coating for brackets was manufactured using a PVD system type (Midas Thermal Evaporator, Vaksis, Ankara, Turkey). Brackets were placed to substrate the e-beam evaporator device by banding base. Electron beam evaporation method was performed at <2 × 10^−6^ Torr vacuum pressure with oil-free pumping for 8 h, and brackets were coated to 1 μ thickness by nanosilver particles.

### Implantation procedure

**Fig. 1:**
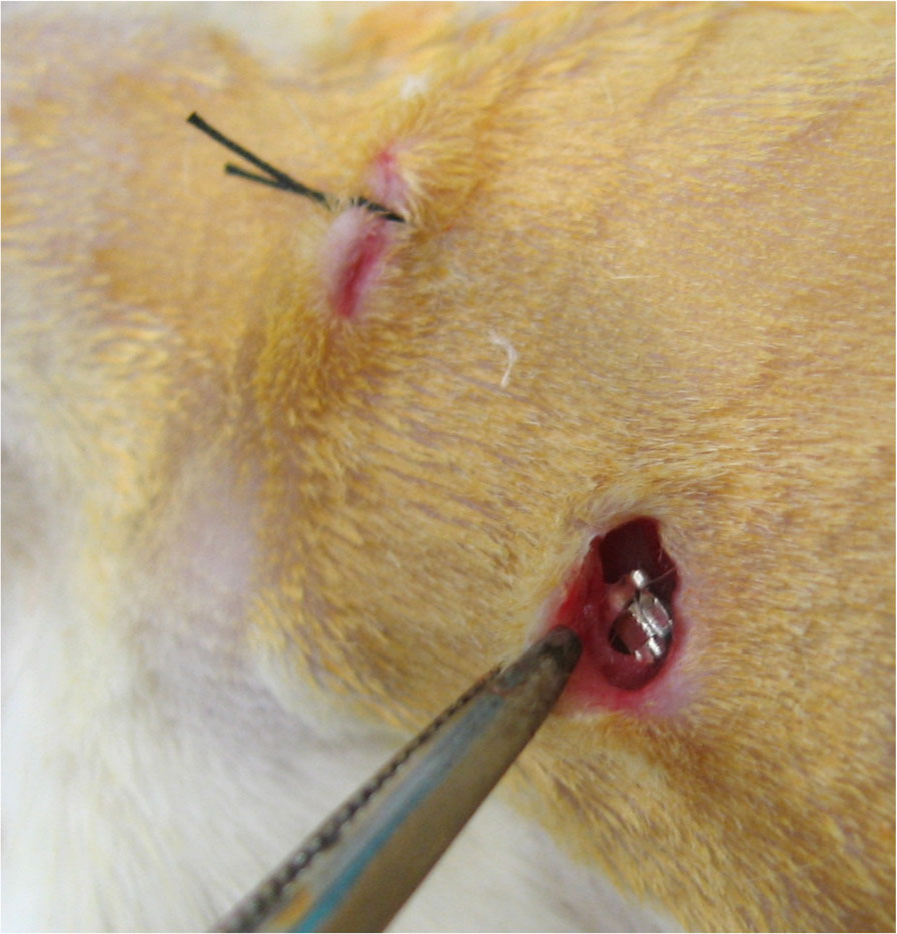
Implantation procedure

A total of 12 healthy female Sprague Dawley rats (mean age and weight 120 days and 200 g) were randomly divided into the study (*n* = 6) and control (*n* = 6) groups. The animals were kept in plastic cages, three animals per cage, with access to food and water ad libitum. After the rats were anesthetized, the dorsal skin was shaved and disinfected. Four different points with the maximum interspace (60 mm) were selected on the back of the rats, and incisions of 5–7 mm long were made with surgical scissors on the dorsum of each rat. For the study and control groups, four nanosilver-coated and four standard brackets were aseptically implanted subcutaneously in the dorsal region of each rat, respectively (Fig. 1). The skin was sutured by only one stitch.

### Collection of the sample

**Fig. 2:**
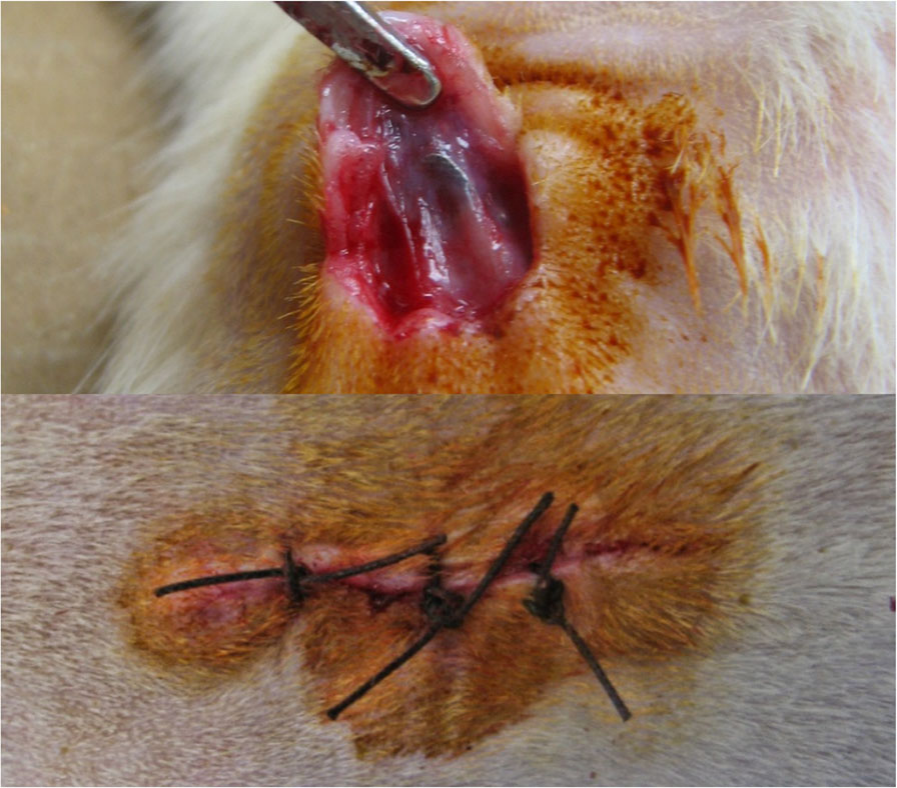
Procedure of taking samples

On days 7, 14, 30, and 60, one implanted bracket and the associated skin and connective tissues were excised from deeply anesthetized rats, and then wounds were resutured (Fig. 2). Samples were immediately fixed in 10 % neutral-buffered formalin. On day 60, all rats were sacrificed by overdose anesthetic solution. The specimens were evaluated for inflammatory response and foreign body reaction.

### Histological evaluation

After the histologic processing, the tissue was serially sectioned longitudinally with a microtome (Leica SM-2000R, Leica Corp., Germany) set at 5–6 μm. The samples were stained with hematoxylin-eosin for histological evaluation using Unna’s method for the evaluation of mast cells. Histological evaluations were made under a light microscope (Nikon Eclipse E-600, Nikon Corp., Japan) at ×40, ×100, ×200, and ×400 magnification. The observer was blinded to the procedure. Evaluation of inflammatory cell and mast cell infiltration was performed according to a previous study [32].

The scoring of the inflammatory cell infiltration is as follows:

- 0 = An absence of inflammatory cells
- 1 = Mild; an average of fewer than 25 inflammatory cells
- 2 = Moderate; an average of 25–124 inflammatory cells
- 3 = Severe; an average of 125 or more inflammatory cells

### Statistical analysis

The statistical significance was determined using the SPSS 20.0 software for Windows (SPSS Inc., Chicago, IL, USA) for mast cell counts. Differences between the study and control groups were tested by the Mann–Whitney *U* test. Values were considered statistically significant at *p* < 0.05.

## Results

After the implantation of the brackets, the animals displayed no behavioral or weight changes or mortality.

### Histological evaluation on day 7

**Fig. 3:**
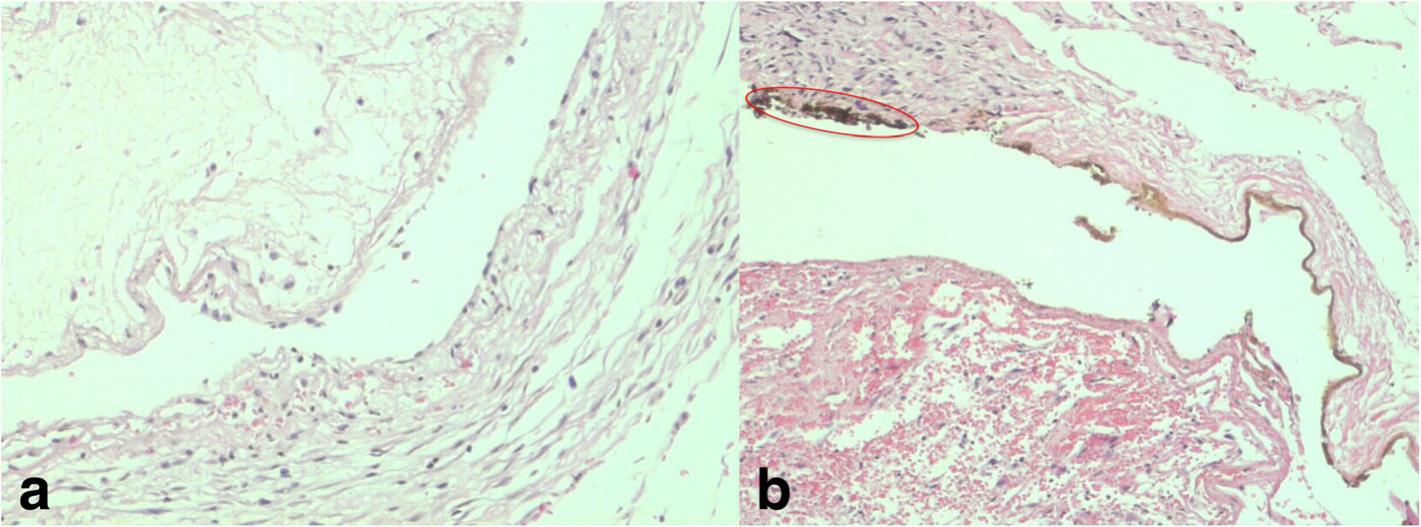
**a** The sample of the control group at day 7. **b** The sample of the study group at day 7, brown-black granules (*encircled*) (H&E; **a**, **b** ×200)

Samples had similar inflammatory reaction in the study and the control groups. In both groups, the incision area was observed to start from the dermis and to extend under the muscle tissue. Exudates including the fibrin and neutrophils were observed on the surface where the epithelial area is damaged. Granulation tissue with rich inflammatory cells, lymphocytes, plasma cells, and the proliferation of the capillary were observed in this area adjacent to the brackets. The brackets were surrounded by exudates and fibrin with hyaline, amorphous structures (Fig. 3a).

The inflammatory score was found as 3 for both groups.

Unlike the control group, in the study group, foreign particles which are brown-black granules were found to be randomly distributed in the granulation tissue adjacent to the bracket. Besides, accumulations of the foreign particles were observed in the study group along the borders of the bracket space (Fig. 3b).

**Fig 4:**
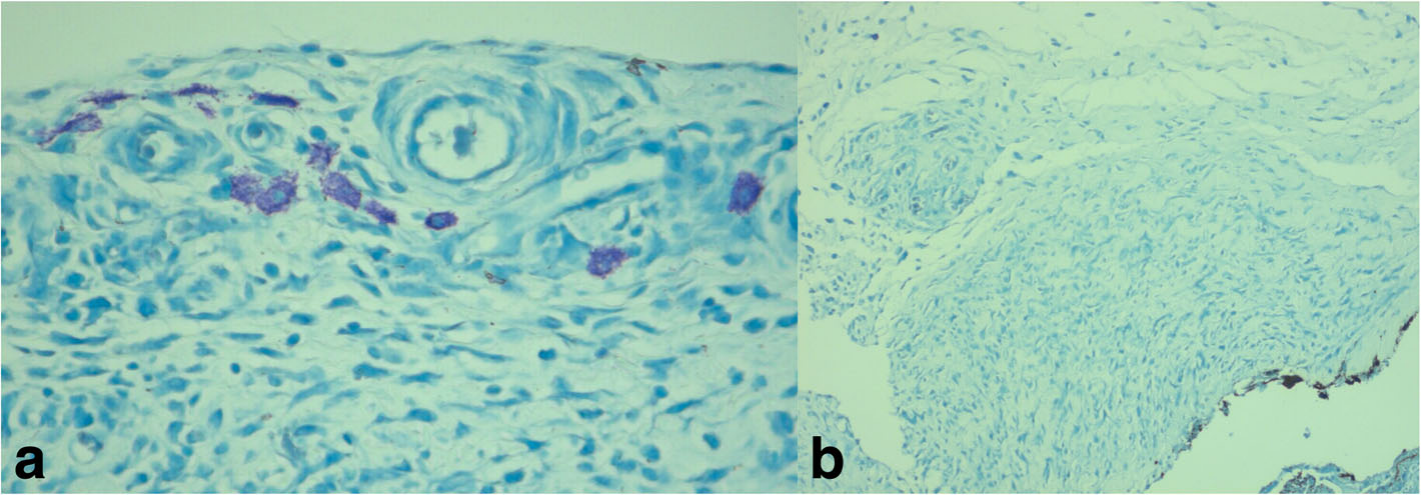
**a** Mast cells of the control group at day 7 (Dominici ×400). **b** Mast cells of the study group at day 7 (Dominici ×200)

Mast cell count was found statistically lower in the study group than in the control group on day 7 (Fig. 4) (Table 1).

**Table 1:** Statistical comparison of groups for mast cell counts

Days	Groups	Mast cell counts				Mann–Whitney *U* test			95 % confidence interval for mean	
		*n*	Mean ± SD	Med.	Min./max.	Mean rank	*U*	*p*	Lower bound	Upper bound
7	Study	6	6.50 ± 4.09	6.50	1.0/11.0	3.83	2.00	0.009*	2.2	10.8
	Control	6	23.83 ± 12.24	21.00	8.0/45.0	9.17			11.0	36.8
	Sum	12	15.17 ± 12.55	11.00	1.0/45.0				12.8	33.6
14	Study	6	23.17 ± 9.91	20.00	14.0/37.0	7.92	9.50	0.180	8.1	22.2
	Control	6	15.17 ± 6.74	14.50	8.0/27.0	5.08			17.876	31.457
	Sum	12	19.17 ± 9.09	16.00	8.0/37.0				13.9	31.4
30	Study	6	24.67 ± 6.47	25.50	17.0/31.0	7.08	14.50	0.589	13.887	20.113
	Control	6	22.67 ± 8.36	21.50	13.0/37.0	5.92			7.3	25.3
	Sum	12	23.67 ± 7.20	22.50	13.0/37.0				2.2	10.8
60	Study	6	17.00 ± 2.97	17.50	13.0/20.0	7.75	10.50	0.240	11.0	36.8
	Control	6	16.33 ± 8.57	13.50	10.0/33.0	5.25			12.8	33.6
	Sum	12	16.67 ± 6.12	16.00	10.0/33.0				8.1	22.2

*p<0.05

### Histological evaluation on day 14

**Fig. 5:**
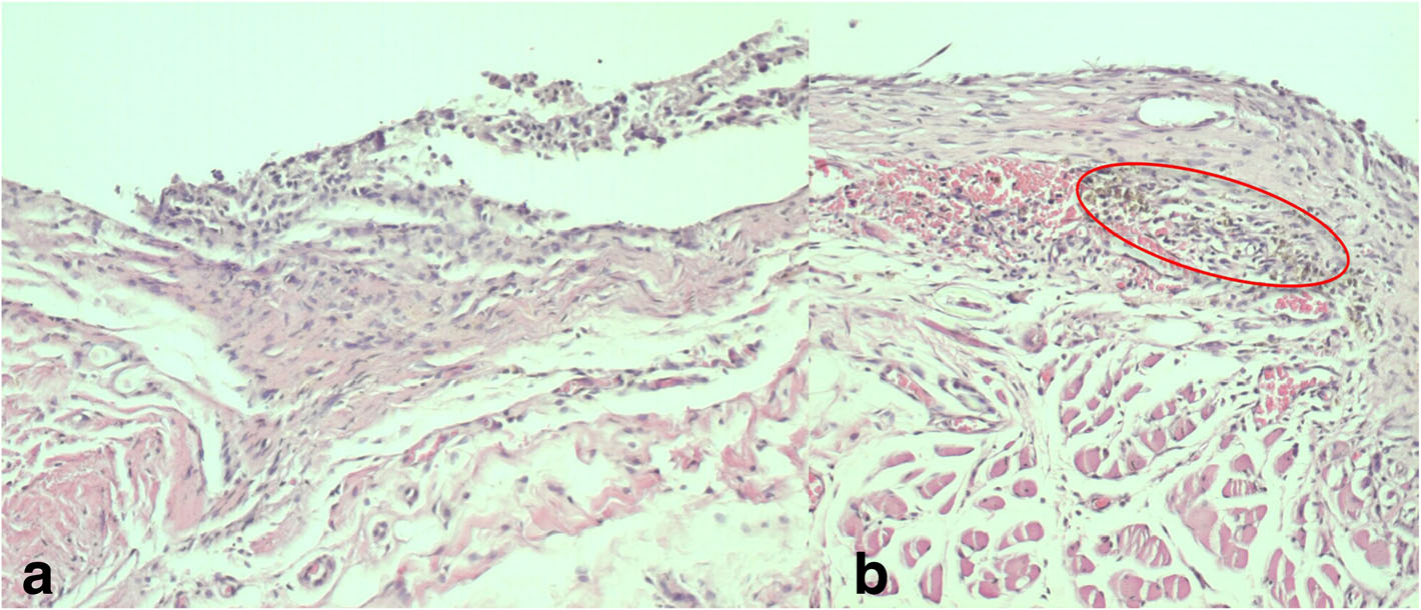
**a** The sample of the control group at day 14. **b** The sample of the study group at day 14 , brown-black granules (*encircled*) (H&E; **a**, **b** ×200)

In all samples in both groups, the incision area was covered with epithelium and improved with fibrous connective tissue including large nucleus active fibroblasts, few inflammatory cells, and large and small capillaries in the section between the epithelium and the bracket (Fig. 5a). It was observed that the intensity of the inflammation on day 17 was less compared to the sample obtained on day 7.

The inflammatory score on day 14 was found as 1.3 for the study and 1.5 for the control group.

Unlike the control group, the foreign particles which are brown-black granules were found to be randomly distributed in the granulation tissue adjacent to the bracket in the study group. It was also observed that the accumulations of foreign particles were present throughout the bracket space (Fig. 5b).

Mast cell counts were not significantly different between the study group and the control group on day 14.

### Histological evaluation on day 30

**Fig. 6:**
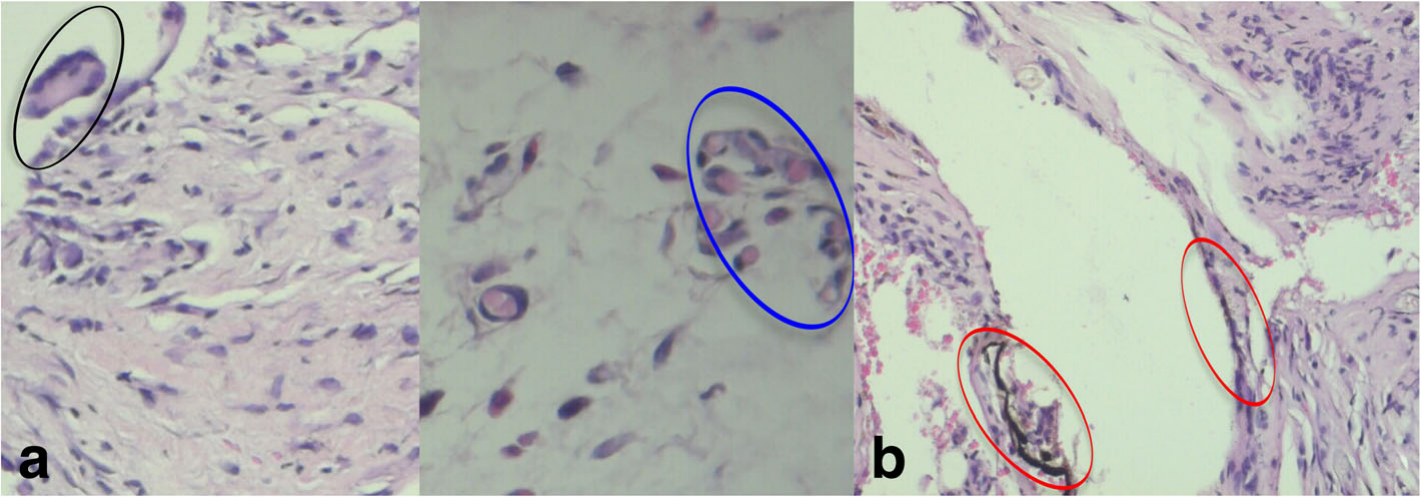
**a** The sample of the control group at day 30, Giant cell (*black encircled*) (H&E; ×200), Eosinophil (*blue encircled*) (H&E, ×400). **b** The sample of the study group at day 30 , brown-black granules (*red encircled*) (H&E; ×200)

In the control group, the incision area was completely closed with epithelium and filled with the connective tissue. In general, a thin fibrous band and collagen connective tissue were observed around the bracket. Mild inflammatory cell infiltration was found in the area under the fibrous band. Unlike the study group, in the control group, a giant cell was observed around the bracket of a sample and eosinophil associated with inflammatory infiltration was seen in another sample (Fig. 6a).

The inflammatory score was found as 1.5 in the control group.

In the study group, the incision area was completely closed with epithelium and filled with the connective tissue. A collagenase thin fibrous band of the connective tissue with foreign particles was observed in the areas adjacent to the bracket. Connective tissues adjacent to the fibrous band were found to be a collagenase structure and including moderate inflammatory cells. Accumulations of foreign particles were found around the vessel wall (Fig. 6b).

The inflammatory score was found to be 0.8 in the study group.

Mast cell counts were not significantly different between the study group and the control group on day 30.

### Histological evaluation on day 60

**Fig. 7:**
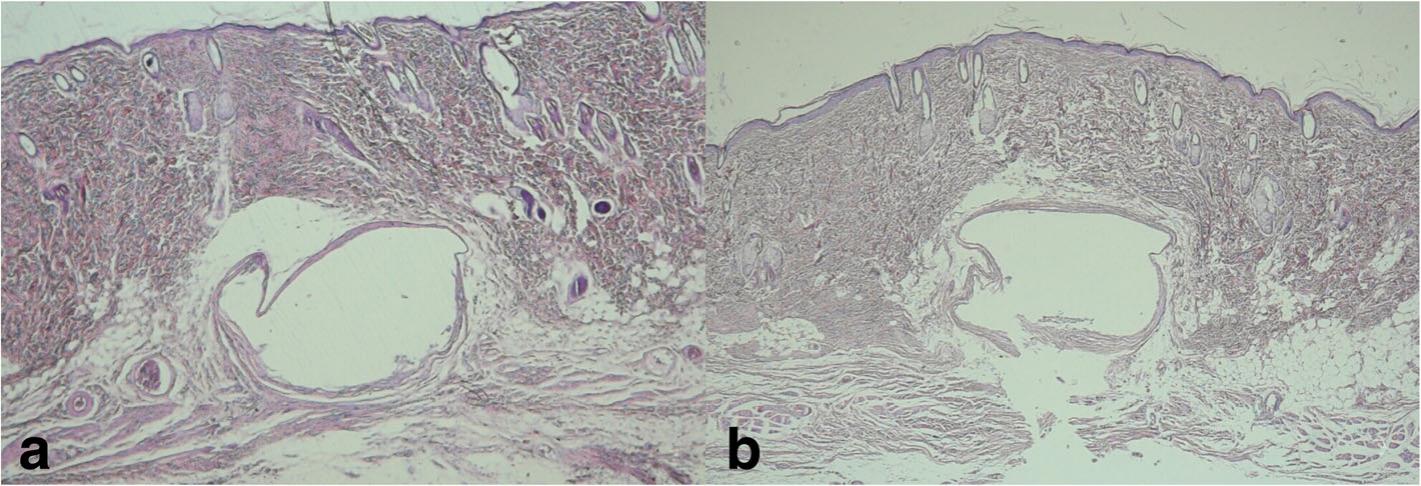
**a** The sample of the control group at day 60 (H&E, ×40). **b** The sample of the study group at day 60 (H&E; ×40)

In both groups, the connective tissue showed no inflammatory reaction. The tissue incision line including the skin layer showed improvement with a morphology similar to that of a healthy tissue. A fibrous band with a few parallel collagen fibers was seen adjacent to the bracket space (Fig. 7a).

The inflammatory score was found as 0 in both groups.

In the study group, foreign particles were observed throughout the bracket space (Fig. 7a).

Mast cell counts were not significantly different between the study group and the control group on day 60.

## Discussion

Biocompatibility test is defined as the ability to create a biological response. Whether an object creates any harmful effects on the body tissues is determined via biocompatibility tests. Most of the materials in contact with the oral tissue, such as the amalgam, are analyzed for biocompatibility by subcutaneous implantation tests. The most commonly used animals in implantation test are mice, rats, rabbits, and guinea pigs [33, 34].

In the present study, the biocompatibility of the nanosilver-coated orthodontic bracket was evaluated by subcutaneous implantation tests since this newly designed orthodontic bracket will be in contact with or placed near the gingiva and oral epithelium during orthodontic treatment. The sample size in animal studies of nanosilver cytotoxicity per group varies considerably in literature [35, 36]. Proper experimental design and statistical analysis of the proposed research project allows the optimum number of animals to be used. Reduction is a rule of the guiding principles underpinning the humane use of animals in scientific research (the three Rs).

In the present study, sample size was calculated by considering a mean difference of mast cell counts between the study group and the control group. With a one-sided significance level of 0.05 and a power of 92 %, a minimum of six animals per group were required. Besides, the value *E* should lie within 10 to 20 for optimum sample size. If a value of *E* is less than ten, then more animals should be included, and if it is more than 20, then the sample size should be decreased (*E* = Total number of animals − Total number of groups) (for the present study, *E* = 12 − 2 = 10) [37]. Although the sample size seems the most important limitation of this study, power analysis and the value *E* support the sample size which is appropriate according to the three Rs.

While the biocompatibility of nanosilver has been shown in many in vitro studies [21, 26, 28], there is only one in vivo study present in the literature [35]. In that study, researchers found that intramuscular implantation of nanosilver and microsilver particles caused serious inflammation reaction and granuloma.

In the present study, the inflammation score was found to be lower compared to the findings of Chen et al. [35]. In the study group of the present study, no symptoms of acute inflammation were observed on day 7 or at the end of the study on day 60, and no granuloma or necrosis was seen during the study. These differing results seem to be related with the different implantation techniques and the areas of application.

In studies in which nanosilver particles were inhaled or exposed by oral administration, no serious side effects on rats [36, 38] were stated.

Macrophages, which comprise 7 % of white blood cells, are formed through the differentiation of monocytes, one of the major components of the immune system. Macrophages provide a defense in protecting the host from damage or infection, and they remove necrosis. In short-term acute inflammation, if the irritants perish from the environment, the macrophages rapidly disappear. In chronic inflammation, macrophage accumulation is continuous. Eosinophil granulocyte and neutrophils also differentiate from white blood cells. Neutrophils are one of the first responders of inflammatory cells to migrate toward the site of inflammation in 6–24 h and return to monocytes within 24–48 h. Eosinophil granulocytes are involved in hypersensitivity reactions and viral infections [39, 40].

In the present study, neutrophils were not seen, except in the area close to the surface epithelium in both groups on day 7. These findings may be an indicator that acute inflammation did not continue till day 7. However, eosinophil granulocytes and giant cells derived from macrophages were observed only in the control group on day 30. These results may indicate that mild symptoms of chronic inflammation and foreign body reaction continued in the control group on day 30.

Therefore, we suggest that nanosilver-coated orthodontic brackets have better tissue compatibility features than standard orthodontic brackets. Mast cell counts, which were found significantly lower in the study group, support these findings.

Currently, nanosilver containing wound dressings with antibacterial properties are used in treatment of burns [7, 12, 13]. These nanosilver dressings have superior features compared to the silver sulfadiazine (SSD) dressings in terms of antibacterial properties, wound healing, shortened time of wound clearing and epithelization, and painless dressing change [7]. These studies noted that nanosilver-containing wound dressings are used safely and highly efficiently for burn patients and they do not show any toxicological side effects. The most common side effects of silver is argyria, and its symptoms are pigmentation in the eyes, face, and skin [41].

In this study, four nanosilver-coated orthodontic brackets were inserted in the dorsal region of each rat in the study group. As a result of our clinical observations, differences considered to be symptoms of argyria were observed in none of the animals. A previous study showed that nanosilver-coated orthodontic brackets prevent caries formation via its antibacterial properties [16]. The present study suggests, with the support of other previous studies, that nanosilver-coated orthodontic brackets have no disadvantage in terms of biocompatibility, and also it can be useful during fixed orthodontic treatment.

## Conclusions

Nanosilver-coated orthodontic brackets were found to have similar properties with the standard type orthodontic bracket in terms of tissue reaction. The nanosilver-coated orthodontic bracket can be a new type of bracket in the field of orthodontics for use in human teeth for the reduction of the areas of tooth decay and demineralization during orthodontic treatment with the advantages of the antibacterial properties of nanosilver. However, the nanosilver-coated orthodontic bracket can be utilized during fixed orthodontic treatment with caution, because the foreign particles which are brown-black granules were found to be randomly distributed in the granulation tissue adjacent to the bracket in the study group. Before the use in humans, further researches are needed with regard to brown-black granules, especially on focusing their deposition of the vessel walls.

## References

[1] Alexander JW (2009). History of the medical use of silver. Surg Infect (Larchmt).

[2] Chwalibog A, Sawosz E, Hotowy A (2010). Visualization of interaction between inorganic nanoparticles and bacteria or fungi. Int J Nanomedicine.

[3] Rai M, Yadav A, Gade A (2009). Silver nanoparticles as a new generation of antimicrobials. Biotechnol Adv.

[4] Kim JS, Kuk E, Yu KN (2007). Antimicrobial effects of silver nanoparticles. Nanomedicine.

[5] Subramani K, Huja S, Kluemper TG, Morford L, Hartsfield Jr JM. Nanotechnology in orthodontics-1: the past, present, and a perspective of the future. In: Subramani K, Ahmed W, Hartsfield JK, editors. Nanobiomaterials in Clinical Dentistry. 1 ed. Amsterdam: Elsevier; 2013. p. 231-45.

[6] Ahn SJ, Lee SJ, Kook JK, Lim BS (2009). Experimental antimicrobial orthodontic adhesives using nanofillers and silver nanoparticles. Dent Mater.

[7] Argirova M, Hadjiiski O (2011). Application of the nanocrystalline silver in treatment of burn wounds in children: InTechOpen.

[8] Kasraei S, Sami L, Hendi S, Alikhani MY, Rezaei-Soufi L, Khamverdi Z (2014). Antibacterial properties of composite resins incorporating silver and zinc oxide nanoparticles on Streptococcus mutans and Lactobacillus. Restor Dent Endod.

[9] Li F, Weir MD, Chen J, Xu HH (2013). Comparison of quaternary ammonium-containing with nano-silver-containing adhesive in antibacterial properties and cytotoxicity. Dent Mater.

[10] Lin S, Huang R, Cheng Y, Liu J, Lau BL, Wiesner MR (2013). Silver nanoparticle-alginate composite beads for point-of-use drinking water disinfection. Water Res.

[11] Quang D, Sarawade P, Jeon S (2013). Effective water disinfection using silver nanoparticle containing silica beads. Appl Surf Sci.

[12] Moiemen NS, Shale E, Drysdale KJ, Smith G, Wilson YT, Papini R (2011). Acticoat dressings and major burns: systemic silver absorption. Burns.

[13] Vlachou E, Chipp E, Shale E, Wilson YT, Papini R, Moiemen NS (2007). The safety of nanocrystalline silver dressings on burns: a study of systemic silver absorption. Burns.

[14] Brutel de la Riviere A, Dossche KM, Birnbaum DE, Hacker R (2000). First clinical experience with a mechanical valve with silver coating. J Heart Valve Dis.

[15] Borzabadi-Farahani A, Borzabadi E, Lynch E (2014). Nanoparticles in orthodontics, a review of antimicrobial and anti-caries applications. Acta Odontol Scand.

[16] Metin-Gursoy G, Taner L, Akca G (2016). Nanosilver coated orthodontic brackets: in vivo antibacterial properties and ion release. Eur J Orthod.

[17] Rosenbloom RG, Tinanoff N (1991). Salivary Streptococcus mutans levels in patients before, during, and after orthodontic treatment. Am J Orthod Dentofacial Orthop.

[18] Gorelick L, Geiger AM, Gwinnett AJ (1982). Incidence of white spot formation after bonding and banding. Am J Orthod.

[19] Bishara SE, Ostby AW (2008). White spot lesion: formation, prevention, and treatment. Semin Orthod.

[20] Julien KC, Buschang PH, Campbell PM (2013). Prevalence of white spot lesion formation during orthodontic treatment. Angle Orthod.

[21] Ryu HS, Bae IH, Lee KG (2012). Antibacterial effect of silver-platinum coating for orthodontic appliances. Angle Orthod.

[22] Moreira DM, Oei J, Rawls HR (2015). A novel antimicrobial orthodontic band cement with in situ-generated silver nanoparticles. Angle Orthod.

[23] Tian J, Wong KK, Ho CM (2007). Topical delivery of silver nanoparticles promotes wound healing. ChemMedChem.

[24] AshaRani PV, Mun GLK, Hande MP, Valiyaveettil S (2009). Cytotoxicity and genotoxicity of silver nanoparticles in human cells. ACS Nano.

[25] Hussain SM, Hess KL, Gearhart JM, Geiss KT, Schlager JJ (2005). In vitro toxicity of nanoparticles in BRL 3A rat liver cells. Toxicol In Vitro.

[26] Park S, Lee YK, Jung M (2007). Cellular toxicity of various inhalable metal nanoparticles on human alveolar epithelial cells. Inhal Toxicol.

[27] Alt V, Bechert T, Steinrucke P (2004). An in vitro assessment of the antibacterial properties and cytotoxicity of nanoparticulate silver bone cement. Biomaterials.

[28] Mohanty S, Mishra S, Jena P, Jacob B, Sarkar B, Sonawane A (2012). An investigation on the antibacterial, cytotoxic, and antibiofilm efficacy of starch-stabilized silver nanoparticles. Nanomedicine.

[29] Acosta-Torres LS, Mendieta I, Nunez-Anita RE, Cajero-Juarez M, Castano VM (2012). Cytocompatible antifungal acrylic resin containing silver nanoparticles for dentures. Int J Nanomedicine.

[30] Ewald A, Gluckermann SK, Thull R, Gbureck U (2006). Antimicrobial titanium/silver PVD coatings on titanium. Biomed Eng Online.

[31] Kilkenny C, Browne WJ, Cuthi I, Emerson M, Altman DG (2012). Improving bioscience research reporting: the ARRIVE guidelines for reporting animal research. Vet Clin Pathol.

[32] Karanth P, Manjunath MK, Roshni, Kuriakose ES (2013). Reaction of rat subcutaneous tissue to mineral trioxide aggregate and Portland cement: a secondary level biocompatibility test. J Indian Soc Pedod Prev Dent.

[33] Sakaguchi RL, Powers JM (2012). Craig’s restorative dental materials.

[34] Anderson JM (2012). Polymer science: a comprehensive reference.

[35] Chen D, Xi T, Bai J (2007). Biological effects induced by nanosilver particles: in vivo study. Biomed Mater.

[36] Ji JH, Jung JH, Kim SS (2007). Twenty-eight-day inhalation toxicity study of silver nanoparticles in Sprague-Dawley rats. Inhal Toxicol.

[37] Charan J, Kantharia ND (2013). How to calculate sample size in animal studies?. J Pharmacol Pharmacother.

[38] Kim YS, Kim JS, Cho HS (2008). Twenty-eight-day oral toxicity, genotoxicity, and gender-related tissue distribution of silver nanoparticles in Sprague-Dawley rats. Inhal Toxicol.

[39] Cotran R, Kumar V, Robbins S (1994). Pathologic basis of disease 5 ed..

[40] Mosser DM, Edwards JP (2008). Exploring the full spectrum of macrophage activation. Nat Rev Immunol.

[41] Trop M, Novak M, Rodl S, Hellbom B, Kroell W, Goessler W (2006). Silver-coated dressing acticoat caused raised liver enzymes and argyria-like symptoms in burn patient. J Trauma.

